# Triglyceride-lowering therapies for hepatic steatosis: mechanisms, efficacy, and clinical perspectives

**DOI:** 10.3389/fphar.2026.1789019

**Published:** 2026-05-12

**Authors:** Seyed Saeed Tamehri Zadeh, Peter P. Toth, Maciej Banach

**Affiliations:** 1 International Lipid Expert Panel (ILEP), Lodz, Poland; 2 Preventive Cardiology, CGH Medical Center, Rock Falls, IL, United States; 3 Ciccarone Center for the Prevention of Cardiovascular Disease, Johns Hopkins University School of Medicine, Baltimore, MD, United States; 4 Faculty of Medicine, John Paul II Catholic University of Lublin, Lublin, Poland

**Keywords:** ANGPTL3, ApoC-III, hepatic steatosis, metabolic dysfunction-associated steatotic liver disease, omega-3 fatty acids, pcsk9, statins, triglyceride

## Abstract

Hepatic steatosis, the hallmark of metabolic dysfunction-associated steatotic liver disease (MASLD), is closely associated with elevated plasma and intrahepatic triglyceride (TG) levels, often driven by insulin resistance, atherogenic dyslipidemia, and metabolic syndrome. Growing evidence suggests that triglyceride-lowering therapies may not only improve systemic lipid profiles but also directly impact hepatic fat accumulation and associated inflammation and fibrosis. This review explores current and emerging TG-lowering therapies, including fibrates, omega-3 fatty acids, pemafibrate, and novel agents such as pegozafermin (an FGF21 analog), and APOC3 and angiopoietin-like protein 3 (ANGPTL3) inhibitors, in the context of hepatic steatosis and MASLD. We discuss the mechanistic rationale behind TG-lowering as a therapeutic strategy, summarize key preclinical and clinical trial findings, and evaluate their effects on liver fat content, liver enzymes, fibrosis markers, and histologic outcomes. While several agents demonstrate promise in reducing intrahepatic fat deposition and improving liver-related outcomes, further large-scale studies are required to establish their long-term efficacy and safety, and potential for disease modification in MASLD/metabolic dysfunction-associated steatohepatitis (MASH). Triglyceride-targeted therapies may offer a valuable adjunct or complementary approach to current treatment paradigms focused on weight loss, insulin sensitization, and antagonizing inflammation.

## Graphical Abstract

**Figure:**
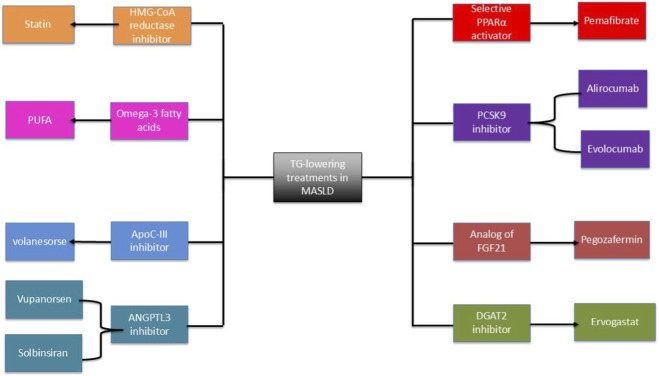

## 1 Introduction

Multinational liver societies from the U.S., Europe, and other regions have agreed to revise the terminology and definitions of non-alcoholic fatty liver disease (NAFLD). This change was driven by concerns that the term “NAFLD” obscures the metabolic origins of the condition and contributes to social stigma. The updated terminology introduces “metabolic dysfunction-associated steatotic liver disease” (MASLD) to replace NAFLD and “metabolic dysfunction-associated steatohepatitis” (MASH) to replace nonalcoholic steatohepatitis. According to the new criteria, MASLD is diagnosed in individuals who have hepatic steatosis along with at least one of five cardiometabolic risk factors, provided that no other underlying causes for liver fat accumulation are identified (Bae, 2024; Rinella et al., 2023).

Several etiologies can lead to hepatic steatosis, including, but not limited to, MASLD, chemotherapy, drugs, alcoholism, and infections (Idilman et al., 2016). A recent meta-analysis of 72 studies from 17 countries demonstrated that the overall prevalence of MASLD is about 32.4% (95% CI 29.9–34.9). The overall incidence of MASLD was estimated to be approximately 46.9 (36.4–57.5) cases per 1,000 person-years. The incidence of MASLD is much higher in men compared with women (70·8 cases per 1,000 person-years and 29·6 cases per 1,000 person-years, respectively) (Riazi et al., 2022).

In most patients, MASLD is correlated with several metabolic comorbidities, including type 2 diabetes, obesity, and dyslipidemia. MASLD increases the risk of CVD, hypertension, cardiomyopathy, heart arrhythmias, and cardiovascular mortality (Kasper et al., 2021). MASLD is also associated with enhanced risk for type 2 diabetes, malignancy, chronic kidney disease and end-stage renal disease, and osteoporosis (Armstrong et al., 2014). Given these clinical attributes, MASLD patients have a higher risk of mortality relative to the general population (Ekstedt et al., 2015). Management of MASLD is important to reduce risk of premature morbidity and mortality.

## 2 Molecular mechanisms for the hepatic steatosis

Lipid accumulation in hepatocytes provokes hepatic insulin resistance (IR) *via* the production of ceramides and diacylglycerol (DAG) (Jornayvaz and Shulman, 2012). Accumulated lipid can also induce lipotoxicity, which is characterized by the development of mitochondrial dysfunction (impaired ATP production and increased biosynthesis of injurious oxygen free radicals by cytochrome oxidase), endoplasmic reticulum (ER) stress and the unfolded protein response, and abnormal autophagy (Neuschwander‐Tetri, 2010). Lipotoxicity can induce immune responses in some liver cells, including stellate cells and Kupffer cells (resident macrophages), contributing to MASH, cirrhosis, and, rarely, hepatocellular carcinoma. Under normal and pathological conditions, TG synthesis and clearance play crucial roles in modulating lipid homeostasis in the liver (Koo, 2013); therefore, understanding the molecular mechanisms behind these pathways constitute reasonable targets for the development of novel therapeutic strategies and treatments.

### 2.1 Fatty acid uptake

Hepatocytes take up plasma free fatty acids (FFAs) which are used for TG synthesis. In the setting of IR there is increased catecholamine release from the central nervous system. These catecholamines bind to beta-adrenergic receptors on the surface of visceral adipocytes, which in turn activate hormone sensitive lipase (HSL) (Arner, 2005). Activation of HSL drives triglyceride lipolysis. In the setting of IR insulin is no longer able to control HSL activity. Hence, increased visceral adiposity is associated with elevations in plasma FFA. A large percentage of this FFA is taken up by the liver, which can process it in multiple ways. These include (Bae, 2024): re-esterifying FFA with glycerol to form triglycerides, which can then be packaged into very low-density lipoprotein (VLDL) and secreted into the central circulation (Rinella et al., 2023); the FFA can be burned as fuel in the mitochondrial matrix *via* beta-oxidation (Idilman et al., 2016); some can be converted to glucose *via* activation of phosphoenolpyruvate carboxykinase; and (Riazi et al., 2022) if these pathways become saturated the excess triglyceride can be deposited as fat within the hepatic parenchyma leading to hepatic steatosis (Bays et al., 2013; Delarue and Magnan, 2007).

Several plasma membrane transporters for the uptake of FFA have been identified, such as, but not limited to, fatty acid binding protein (FABP), fatty acid transporter protein (FATP), fatty acid translocase (FAT)/CD36, and the caveolins. FABP contains several isoforms and overexpression of FABP 4 and FABP5 were detected in patients suffering from hepatic steatosis and IR (Westerbacka et al., 2007). Deletion of the gene for FABP1 inhibits hepatic steatosis (Newberry et al., 2003). FATP inhibition is also associated with reduced hepatic TG accumulation *in vivo* (Doege et al., 2006). FAT/CD36 promotes hepatic FFA uptake. Its expression is increased by diet-induced obesity and correlates with increased TG mass in the liver (Greco et al., 2008). The caveolins play a role in the formation of intracellular lipid droplets as well as cell signaling and division, suggesting that the caveolins are also important for liver regeneration (Fernández et al., 2006).

### 2.2 *De novo* lipogenesis

In addition to increased adipocyte-derived FFA uptake, *de novo* lipogenesis (DNL) can also promote hepatic steatosis (Donnelly et al., 2005). DNL helps to convert excess amounts of carbohydrates and amino acids to fatty acids. During the first step of DNL, ATP-citrate lyase (ACLY) catalyzes the conversion of citrate and coenzyme A (CoA) to acetyl-CoA and oxaloacetate. Acetyl-CoA carboxylase (ACC) carboxylates acetyl-CoA to malonyl-CoA; additional residues of acetyl CoA can then be added to produce successively longer fatty acid chains. Fatty acid synthase (FAS) catalyzes the last step of DNL (Alves-Bezerra and Cohen, 2017). The expression of FAS and ACC is increased in patients with MASLD. ACC inhibition was shown to be associated with decreased hepatic TG mass *in vivo*, by both stimulating fatty acid oxidation as well as suppressing fatty acid biosynthesis (Abu-Elheiga et al., 2005). Interestingly, whole body inhibition of FAS and ACC contributed to embryonic death (Chirala et al., 2003). On a zero-fat diet, liver-specific inhibition of FAS precipitates hypoglycemia and fatty liver, which are mitigated following the administration of dietary fat (Chakravarthy et al., 2005).

The expression of FAS and ACC can be induced by two main transcriptional factors, namely, carbohydrate response element binding protein (ChREBP) and sterol regulatory element binding protein 1c (SREBP-1c). These transcription factors are overexpressed in mice with MASLD (Postic and Girard, 2008). In contrast, only enhanced expression of SREBP-1c is observed in humans with MASLD (Higuchi et al., 2008).

DNL suppression is associated with reduced generation and secretion of VLDL-TG *in vivo*; nevertheless, approximately 5% of VLDL-TG contains FAA originating from DNL (Diraison et al., 2003). FAA-derived DNL incorporation into VLDL-TG can be enhanced in different conditions, such as infections, high-carbohydrate diet, and alcohol intake, which was shown to be positively associated with enhanced plasma levels of VLDL-TG. Increased DNL can be detected in obese individuals (Strable and Ntambi, 2010) and patients with MASLD (Ferre and Foufelle, 2010). Therefore, FAA-derived DNL can partly explain increased levels of plasma TG.

### 2.3 TG and insulin resistance

**FIGURE 1:**
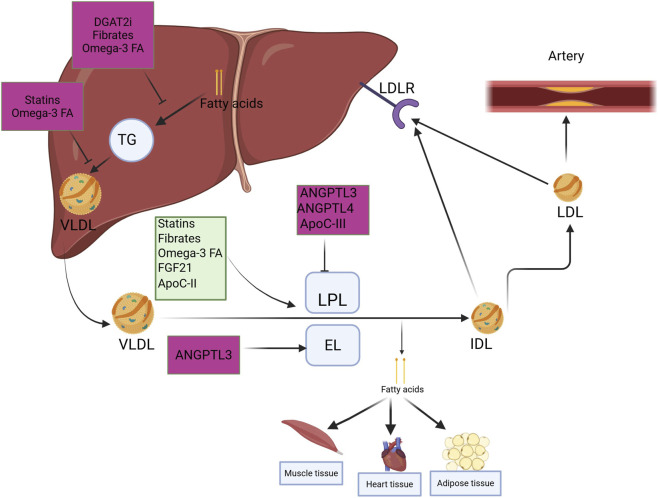
Metabolism of triglycerides, based on the information from Filtz et al. (2024)- CC BY license - permission not required. In the liver, free fatty acids are esterified into triglycerides (TGs) and incorporated into VLDL, which are then secreted into the circulation. This process can be attenuated by DGAT2 inhibitors, which directly block TG synthesis, as well as by fibrates and omega-3 fatty acids, which reduce hepatic TG availability. Statins indirectly decrease VLDL production by limiting cholesterol-dependent ApoB assembly, while omega-3 fatty acids also suppress TG incorporation into VLDL and its secretion. Upon entering the bloodstream, VLDL undergoes hydrolysis predominantly by LPL, with a lesser contribution from EL, leading to the release of FFA from the core TGs and surface phospholipids. The activity of LPL is inhibited by apoC-III, ANGPTL3, and ANGPTL4, while it is activated by apoC-II, statins, fibrates, omega-3 fatty acids, and FGF21 activators. ANGPTL3 also inhibits EL activity. When a substantial proportion of TGs in VLDL is hydrolyzed, VLDL is converted into VLDL remnants IDL, approximately half of which are taken up by the liver, while the remainder is further metabolized into LDL. LDL can bind to LDL receptor and be taken up by the liver. LDL can deposit in the blood vessel walls, forming atherosclerotic plaques. A*bbreviations: TG, triglyceride; VLDL, v*ery-low-density lipoprotein; DGAT, diacylglycerol O-acyltransferase LPL, lipoprotein lipase; EL, endothelial lipase; ApoC-II, Apolipoprotein CII; ApoC-III, Apolipoprotein C-III; FGF21, Fibroblast growth factor 21; IDL, intermediate-density lipoproteins; LDL, Low-density lipoprotein. Figure was created with BioRender.com.

Hypertriglyceridemia, a key characteristic of IR and metabolic syndrome, arises from two primary metabolic disruptions (Bae, 2024): increased hepatic production of VLDL particles that are rich in TGs; and (Rinella et al., 2023) reduced hydrolysis of TGs from VLDL and chylomicrons in the bloodstream, due to diminished activity of lipoprotein lipase (LPL). LPL function declines when levels of apo CII, a necessary cofactor, are lowered, and apo CIII, which inhibits LPL, is elevated (Jong et al., 1999). Additionally, LPL activity is further inhibited by angiopoietin-like protein 3 (ANGPTL3) and Angiopoietin-like protein 4 (ANGPTL4), the latter of which inactivates LPL by converting its active dimeric form into inactive monomers (Köster et al., 2005; Lichtenstein et al., 2007). LPL activity can also be enhanced by statins, fibrates, omega-3 fatty acids, and fibroblast growth factor 21 (FGF21) activators, which collectively promote TG hydrolysis (Figure 1). As a result, blood concentrations of chylomicron remnants, VLDL, and partially degraded VLDL particles, along with TGs, become elevated. Impaired chylomicron breakdown also reduces the release of surface components containing apo A-I, thereby decreasing apo A-I availability and compromising HDL formation. Moreover, as VLDL particles accumulate, activity of cholesteryl ester transfer protein (CETP) increases. CETP facilitates a 1:1 M exchange of triglycerides from VLDL for cholesteryl esters in HDL. As HDLs become enriched with triglycerides, they are more readily hydrolyzed by hepatic lipase. This increased lipolysis makes HDLs unstable and leads to dissociation of apoAI. The freed apoAI may be taken up and degraded by the megalin-cubilin-amnionless complex in the kidney’s proximal tubules, contributing to further reductions in HDL-C. Additionally, IR adipose tissue has a more direct role in altering HDL metabolism. As fat tissue expands and becomes resistant to insulin, it becomes infiltrated by macrophages that generate a pro-inflammatory environment. Cytokines released in this context suppress the expression of several key cholesterol transport proteins, including ATP-binding cassette transporter A1 (ABCA1), ATP-binding cassette subfamily G member 1 (ABCG1), and scavenger receptor BI (SR-BI), which reduces HDL lipidation and speciation (Bays et al., 2013; Zhang et al., 2010).

### 2.4 TG secretion and metabolism

TGs are hydrophobic and require a carrier vehicle in order to be distributed to systemic tissues where they are oxidized as fuel. Apolipoprotein B (apoB) 100 is the scaffold for the assembly of nascent VLDL particles in the liver. At the first step of VLDL assembly, pre-VLDL forms during the translation of apoB100 in the rough ER; thereafter, pre-VLDL matures in the Golgi and can be secreted from the liver through exocytosis. The frequency of fatty liver is increased in those with mutations in apoB100, highlighting the importance of this glycoprotein in hepatic lipoprotein metabolism (Tanoli et al., 2004).

There is a well-known association between VLDL secretion and diabetes. IR causes enhanced liver TG content due to enhanced DNL and increased FFA uptake. Enhanced rates of VLDL secretion are observed in obese individuals with MASLD (Fabbrini et al., 2010a). Importantly, the levels of liver TG and VLDL secretion do not increase in parallel and there is a limitation for VLDL secretion (Fabbrini et al., 2008). Genetic mutations in apoB100 are associated with both decreased rates of VLDL secretion and hepatic steatosis (Welty, 2014). Mipomersen decreases plasma values of LDL by suppressing VLDL secretion; however, it can cause hepatic steatosis (Visser et al., 2010). Mutations in transmembrane 6 superfamily member 2 (TM6SF2) cause hepatic steatosis and reduced VLDL secretion (Smagris et al., 2016).

The principal means by which the liver stores and exports FAA is by the formation of TG. In normal situations, the majority of TG exports to adipose tissue and skeletal muscle in the form of VLDL and a small amount of TG is stored in the liver. In the setting of MASLD, liver TG accumulation can lead to lipotoxicity; however, some evidence suggests that enhanced liver VLDL secretion and TG storage can provide some protection against FAA-induced hepatotoxicity (Day and James, 1998). Listenberger et al. noted that unsaturated fatty acids (UFAs) act as protective factors *via* stimulating liver TG accumulation (Listenberger et al., 2003). UFAs, both endogenous and exogenous, are readily incorporated into lipid droplets, allowing for safe lipid storage without causing cellular toxicity. In contrast, saturated fatty acids (SFAs) are more difficult to store and can induce hepatocyte apoptosis (Listenberger et al., 2003; Mantzaris et al., 2011). However, when UFAs are present alongside SFAs, they promote the conversion of SFAs into TGs and their incorporation into lipid droplets, reducing their toxic effects (Listenberger et al., 2003; Mantzaris et al., 2011). Notably, UFAs can also become toxic to hepatocytes if TG synthesis is impaired, as this prevents safe lipid storage (Listenberger et al., 2003). Preclinical studies have shown that diacylglycerol acyltransferase 2 (DGAT2) inhibition significantly reduces both hepatic and circulating TG levels. DGAT2, an ER–resident enzyme that catalyzes the final step of TG synthesis and can also localize to lipid droplets, plays a central role in hepatic lipid storage. Mechanistically, its inhibition suppresses TG synthesis and redirects diacylglycerol toward phospholipid production, leading to attenuation of DNL *via* reduced activation of SREBP-1c (Choi et al., 2007; Li et al., 2015; Yu et al., 2005). However, DGAT2 inhibition also impairs the esterification of excess fatty acids into inert TG, resulting in the accumulation of lipotoxic intermediates such as FFAs, diacylglycerols, and ceramides. These species induce hepatocellular stress through oxidative stress, ER stress, and mitochondrial dysfunction, thereby promoting inflammation, cell death, and fibrogenesis (Bu, 2023; Yamaguchi et al., 2007).

## 3 Dyslipidemia and MASLD

IR and dyslipidemia play important roles in the pathogenesis of MASLD. Insulin modulates the metabolism of lipid*s via* inhibiting the synthesis of VLDL and enhancing TG storage in adipose tissue. Moreover, insulin can affect the activity of LPL, which participates in the lipolysis of VLDL. In the setting of IR, fat storage in adipose tissue is decreased, leading to enhanced accumulation of FFA in the liver, which induces fibrotic and inflammatory pathways and, as a result, promotes the progression of MASLD to MASH or cirrhosis (Lonardo et al., 2021). FFAs can also act as a substrate for the synthesis of VLDL triglycerides and phospholipids, which results in higher serum levels of ApoB in the setting of MASLD. These VLDL particles undergo lipolysis mediated by LPL to form intermediate density lipoprotein (IDL) and subsequently low-density lipoprotein (LDL). Hepatic lipase can further act on triglyceride-enriched LDL, converting them into small dense LDL (sdLDL) particles, which are considered highly atherogenic. Additionally, in patients with MASLD, reduced levels of HDL can be observed that can reduce reverse cholesterol transport and contribute to endothelial dysfunction. In IR, HDL particle levels are reduced due to impaired production and increased clearance. Expression of apolipoprotein A-I (ApoA1) and ABCA1 is decreased in the jejunum, adipose tissue, and liver, limiting nascent HDL formation. This is partly because the ApoA1 gene contains an insulin response element, making it susceptible to downregulation by hyperinsulinemia. In addition, the transfer of surface components from chylomicrons to HDL is reduced, further limiting HDL maturation. Finally, hepatic lipase activity is increased, promoting the catabolism of triglyceride-rich HDL particles and accelerating HDL clearance (Annema and von Eckardstein, 2016; Florentin et al., 2008).

## 4 Why is hypertriglyceridemia important?

The American College of Cardiology/American Heart Association (ACC/AHA) guidelines acknowledged high levels of triglyceride (TG) as a major risk factor for cardiovascular diseases (CVDs) (Arnett et al., 2019). Hypertriglyceridemia is a component of metabolic syndrome, a well-known constellation of risk factors for CVD (Rosenzweig et al., 2019). It has been shown that those with hypertriglyceridemia and increased waist circumference are at high risk for CVD related events (Miller et al., 2011).

There are well-established correlations between several CVD risk factors, such as obesity, HDL-C values, elevated levels of TG, sedentary behaviours, and diabetes, which make the role of hypertriglyceridemia in CVD complex. A meta-analysis of over 300,000 individuals demonstrated that the association between TG levels and CVD was mitigated following adjustment for non-HDL-C and HDL-C (Assessment, 2009). Another meta-analysis of 35 observational studies revealed a significant association between fasting TG and enhanced risk of CVD and cardiovascular mortality after adjustment for well-known CVD risk factors (Murad et al., 2012). While new guidelines suggest clinicians take into account hypertriglyceridemia as a major risk factor for CVD, no cardiovascular benefits regarding triglyceride lowering with drugs have as yet been reported (Simha, 2020).

The main lipid determinants of residual CV risk after risk stratified LDL-C goal attainment are lipoprotein(a), TG-rich lipoproteins (TRL) such as VLDL remnants and IDL, and inflammation (Gomez-Delgado et al., 2023). Sergio et al. evaluated the association between serum values of TG and subclinical noncoronary atherosclerosis in healthy subjects and found a significant association between subclinical atherosclerosis and TG ≥ 150 mg/dL, which remained significant in both those with elevated LDL-C levels and normal LDL-C levels. Moreover, TG ≥ 150 mg/dL had a significant association with arterial inflammation (Raposeiras-Roubin et al., 2021). These findings highlight the potential of targeting hypertriglyceridemia in the primary prevention of CVD.

Severe hypertriglyceridemia is clinically important because it can induce pancreatitis *via* the cytotoxic and proinflammatory effects of FFA on vascular endothelial cells and pancreatic acinar calls (Guo et al., 2019). A meta-analysis of 927,218 patients revealed a significant association between fasting TG and pancreatitis with an odds ratio (OR) of 3.96 [95% CI, 1.27–12.34] (Murad et al., 2012).

## 5 TGs and hepatic steatosis

Hypertriglyceridemia is known as the most prevalent type of dyslipidemia in patients suffering from MASLD (Sahebkar et al., 2014). Available data have also demonstrated a higher risk of MASLD in patients with hypertriglyceridemia. Eguchi et al. sought to identify the metabolic risk factors of MASLD among the Japanese general population and found a higher prevalence of MASLD in patients with TG > 150 mg/dL (1.7 mmol/L) (Eguchi et al., 2012). Another cross-sectional study among 14,251 Japanese general population detected a higher odds of MASLD (OR: 2.345 [95%CI: 2.000 to 2.749]) for those with TG levels ≥150 mg/dL (1.7 mmol/L) (Hu et al., 2022). The positive association between TG levels and incident MASLD was also reported in several prospective cohort studies (Kitae et al., 2019; Zheng et al., 2018). Recent evidence indicates that hypertriglyceridemia is independently associated with MASH in patients with type 2 diabetes (Castera et al., 2023). A study of 307 nonobese patients with MASLD demonstrated that MASH was more prevalent in patients with elevated TG levels compared with those with normal TG, and that elevated TG levels were independently associated with advanced liver fibrosis (OR 1.044, 95% CI: 1.005–1.083) (Leung et al., 2017). Increases in TG levels were also independent predictors of advanced liver fibrosis in patients with MASH (Méndez-Sánchez et al., 2020). Therefore, effort has been made to manage hepatic steatosis using TG-lowering medications.

## 6 Conventional TG-lowering treatments

### 6.1 Statins

According to ACC/AHA recommendations, initiating or intensifying statin therapy is indicated for individuals with moderate hypertriglyceridemia who are at moderate risk for atherosclerotic cardiovascular disease (ASCVD) (Grundy et al., 2018). Similarly, patients with severe HTG and moderate ASCVD risk should also be considered for statin therapy (Grundy et al., 2018). In alignment with this, the European Society of Cardiology/European Atherosclerosis Society (ESC/EAS) guidelines advise statin use (plus other non-statin drugs for hypercholesterolemia) in high-risk ASCVD patients with TG levels exceeding 200 mg/dL (2.3 mmol/L) (Banach et al., 2021; Mach et al., 2019). Statins primarily reduce overall ASCVD risk by significantly lowering LDL-C and exerting various beneficial effects, including improvements in endothelial function and reductions in oxidative stress, plaque instability, and inflammation (Oesterle et al., 2017). Statin monotherapy typically reduces LDL-C by 25%–55%, depending on the dose (Karlson et al., 2016), but their effect on triglycerides is more modest, generally lowering levels by 15%–30% through reduced VLDL production and enhanced clearance (Karlson et al., 2016). There are some available data that pitavastatin may exert more beneficial effect on TG (>30%) and has the stronger effect on HDL cholesterol in comparison to other statins. By reducing cell proliferation in the liver as well as by inhibiting the phosphatidyl-inositol 3-kinase (PI3K), pitavastatin may also reduce the risk of new onset diabetes, which may be beneficial in patients with atherogenic dyslipidemia (in the course of obesity, diabetes, and metabolic syndrome) (Banach et al., 2022; Sahebkar et al., 2021; Seo et al., 2022; Thakker et al., 2016). Only 20%–50% of patients with baseline TG ≥ 177 mg/dL (≥2 mmol/L) achieve TG < 150 mg/dL (<1.7 mmol/L) on statin therapy, indicating the need for additional triglyceride-lowering treatments (Karlson et al., 2016). Other LDL-C-lowering therapies, such as ezetimibe, proprotein convertase subtilisin/kexin type 9 (PCSK9) inhibitors, bempedoic acid, or obicetrapib, are not specifically indicated for HTG, as their primary goal is to address LDL-C and overall ASCVD risk (Mach et al., 2019).

### 6.2 Fibrates

Fibrates mainly impact TG levels and to a lesser extent, enhance the levels of HDL-C and, in the case of fenofibrate, can reduce levels of LDL-C. Fibrates exert their lipid-lowering effects by activating peroxisomal proliferator-activated receptor alpha (PPARα), which results in increased expression of LPL, fatty acid transport and binding proteins, carnitine palmitoyltransferase II, medium- and long-chain acyl-CoA dehydrogenase, peroxisomal FA β-oxidation, and acyl-CoA oxidase. Fibrates also enhance hepatic insulin sensitivity through PPARα-related acid β-oxidation and overexpression of FGF21 (Kostapanos et al., 2013; Tanaka et al., 2017). With these properties they have shown some capacity for reduction of micro- and macrovascular complications in patients with diabetes (Averna et al., 2021; Banach et al., 2024).

### 6.3 Omega-3 fatty acids

Omega-3 fatty acids, including alpha-linolenic acid (ALA) from plants and eicosapentaenoic acid (EPA)/docosahexaenoic acid (DHA) from fish or supplements, have been investigated for reducing residual CVD risk in statin-treated patients by lowering triglycerides, stabilizing plaques, reducing inflammation, and inhibiting platelet aggregation (Yokoyama et al., 2022).

The open-label Japan EPA Lipid Intervention Study (JELIS) trial evaluated 18,645 patients with hypercholesterolemia (with or without coronary heart disease) and found that adding 1,800 mg of EPA daily to statin therapy resulted in a 19% relative reduction in major cardiovascular events over 4.6 years, independent of LDL-C reduction (Yokoyama et al., 2007). Subgroup analysis revealed that the overall benefit in the JELIS trial was primarily due to a significant 19% relative risk reduction in the secondary prevention group, while no statistically significant effect was observed in the primary prevention group (Yokoyama et al., 2007). In contrast, the ASCEND trial (15,480 diabetic patients without CHD) (Group ASC et al., 2018) and the VITAL trial (25,871 healthy middle-aged adults) (Manson et al., 2019) did not show significant reductions in overall cardiovascular events with omega-3 supplementation (840 mg/day and 1g/day, respectively). However, VITAL did show a 28% reduction in total myocardial infarction (Manson et al., 2019). Substudies like VITAL-CKD and VITAL Rhythm also found no benefit in preventing chronic kidney disease progression or atrial fibrillation development (Albert et al., 2021; de Boer et al., 2019).

The STRENGTH trial in high-risk, statin-treated patients with elevated TGs was stopped early due to no difference in major cardiovascular events between omega-3 and placebo (Bhatt et al., 2019; Nicholls et al., 2020). REDUCE-IT demonstrated that icosapent ethyl (pure EPA) reduced cardiovascular events by 25% and cardiovascular mortality by 20% in high-risk statin-treated patients with elevated TGs, independent of TG lowering, and is the only non-statin therapy proven to reduce ASCVD mortality (Bhatt et al., 2019).

Based on these findings and numerous *post hoc* analyses, the ESC/EAS recommends IPE for high-risk patients on statins with TGs of 135–500 mg/dL (Mach et al., 2019). IPE is the only TG-targeted nonstatin therapy approved by the U.S. Food and Drug Administration (FDA) to reduce ASCVD risk, indicated as an add-on to maximally tolerated statins in adults with TGs ≥150 mg/dL and either established CVD or diabetes with ≥2 additional risk factors, and also approved to lower TGs in severe hypertriglyceridemia (≥500 mg/dL) (Virani et al., 2021). However, IPE is unavailable in many countries, where high-dose EPA/DHA (2–4 g/day) is recommended instead (Banach et al., 2024; Solnica et al., 2024).

### 6.4 PCSK9 inhibitors

Serum PCSK9 levels influence the metabolism of TRLs derived from both intestinal and hepatic sources (Dijk et al., 2018). Multiple studies have shown a modest positive correlation between PCSK9 concentrations and TG levels (Baass et al., 2009; Brouwers et al., 2011; Lakoski et al., 2009). Individuals with PCSK9 gain-of-function mutations tend to have elevated levels of VLDLs and remnant lipoproteins (Lalanne et al., 2005; Ouguerram et al., 2004). Moreover, PCSK9 levels are closely associated with IDLs, which are products of VLDL lipolysis, further supporting its role in TRL metabolism (Guardiola et al., 2015; Kwakernaak et al., 2014). PCSK9 inhibitors significantly lower remnant lipoproteins (Toth et al., 2016; Toth et al., 2018). Elevated serum PCSK9 concentrations have also been linked to higher TG levels in patients with proteinuria and chronic kidney disease, conditions often characterized by TRL abnormalities.

Treatment with PCSK9 inhibitors provide modest reductions in TG levels. For instance, the FOURIER trial demonstrated that evolocumab significantly decreased serum TGs compared with placebo (−16.7% vs −0.7%). These reductions are thought to result from multiple mechanisms influencing both the production and clearance of TRLs. Primarily, the marked increase in LDL receptor activity induced by PCSK9 inhibitors enhances the clearance not only of LDL particles but also the entire spectrum of VLDL species and IDL. PCSK9 inhibitors likely do this by upregulating the expression of other receptors involved in hepatic lipoprotein clearance, such as VLDL receptors, apoE2 receptors, LDL receptor-related protein, and CD36 (platelet glycoprotein 4) (Druce et al., 2015).

## 7 Emerging TG-lowering therapies

### 7.1 Targeting apolipoprotein C-III (apo-CIII)

Apolipoprotein C-III (apo-CIII), a glycoprotein comprised of 79 amino acids and primarily produced by hepatocytes, is located on the surface of chylomicrons, HDL, and VLDL (Ramms and Gordts, 2018). It was demonstrated that apoC-III enhances plasma TG values *via* different mechanisms, including inhibiting TG removal from the circulation, reducing TG hydrolysis by inhibiting LPL, and enhancing VLDL secretion into the blood circulation (Spagnuolo and Hegele, 2023). Furthermore, decreased values of TG and reduced risk of CVD events were observed in those with loss-of-function mutations in apoC-III (Jørgensen et al., 2014); as a result, several drugs with the ability to inhibit apoC-III have been developed using different approaches, such as an antisense oligonucleotide (ASO) and small interfering RNA (siRNA) with the aim of managing hypertriglyceridemia (Table 1). ApoC-III can be inhibited through direct or indirect approaches. Direct inhibitors like ASOs (volanesorsen, olezarsen) and siRNAs (plozasiran) target and degrade ApoC-III mRNA in hepatocytes, leading to potent and selective reductions in protein levels and improved triglyceride metabolism (Biolo et al., 2025; Taskinen and Borén, 2016). In contrast, indirect inhibitors such as fibrates, omega-3 fatty acids, and lifestyle changes modulate upstream pathways that influence ApoC-III expression (Taskinen et al., 2019).

**TABLE 1:** Clinical trials of drugs targeting APOC-III.

Trial	Year	Drug	Population	TG reduction	Other lipid changes
APPROACH, phase 3 (Witztum et al., 2019)	2019	Volanesorsen	66 patients with FCS, TG ≥ 750 mg/dL	76.5% decrease at 3 months with 300 mg	APOC-III: 84.2% decrease Non-HDL-C: 45.9% decrease ApoB: 19.5% increase LDL-C: 135.6% increase HDL-C: 46.1% increase
COMPASS, phase 3 (Gouni-Berthold et al., 2021)	2021	Volanesorsen	114 patients with MCS or FCS with TG ≥ 500 mg/dL	71.2% decrease at 3 months with 300 mg	APOC-III: 76.1% decrease Non-HDL-C: 27.3% decrease ApoB: 5.8% increase LDL-C: 95.5% increase HDL-C: 61.2% increase
Tardif et al., phase 2 (Tardif et al., 2022)	2022	Olezarsen	114 patients with TG 200–500 mg/dL and established ASCVD or high ASCVD risk	60% decrease with 50 mg at 6 months	APOC-III: 74% decrease Non-HDL-C: 19% decrease ApoB: 12% decrease LDL-C: 3% increase HDL-C: 29% increase
Bridge–TIMI 73a, phase 2 (Bergmark et al., 2024)	2024	Olezarsen	154 patients with TG 150–499 mg/dL and increased ASCVD risk, or TG ≥ 500 mg/dL	53.1% decrease with 80 mg at 6 months	APOC-III: 73.2% decrease Non-HDL-C: 23.1% decrease ApoB: 18.5% decrease LDL-C: 7.7% increase HDL-C: 39.6% increase
Balance, phase 3 (Stroes et al., 2024)	2024	Olezarsen	66 patients with FCS	43.5% decrease with 80 mg at 6 months	APOC-III: 73.7% decrease Non-HDL-C: 24.2% decrease ApoB: 84% decrease
Essence–TIMI 73b, phase 3 (Marston et al.)	2026	Olezarsen	468 patients with TGs ≥150 mg/dL, presence or high risk for cardiovascular disease, and non-calcified plaque	63.9% decrease with 80 mg at 6 months	Remnant cholesterol: 71.9% decrease ApoB: 16% decrease LDL-C: 2% decrease
Essence–TIMI 73b, phase 3 (Wang et al., 2026)	2026	Olezarsen	1,349 patients on olezarsen, including 309 receiving concomitant fibrate therapy	Olezarsen and fibrate: 76.6% decrease with 80 mg at 12 months Olezarse: 49.5% decrease with 80 mg at 12 months	APOC-III, non-HDL-C, and total cholesterol were also greater in fibrate users than non-users.
SHASTA-2 Phase 2 (Gaudet et al., 2024)	2024	Plozasiran	226 patients with TG 500–4,000 mg/dL	74.2% decrease with 50 mg at 6 months	APOC-III: 78.3% decrease Non-HDL-C: 26.9% decrease ApoB: 18.3% decrease LDL-C: 10.4% decrease HDL-C: 50.5% increase RC: 52.8% decrease
MUIR, phase 2 (Ballantyne et al., 2024)	2024	Plozasiran	353 patients with TG 150–499 mg/dL and LDL-C ≥ 70 mg/dL or non-HDL-C ≥ 100 mg/dL	74.2% decrease with 50 mg at 6 months	APOC-III: 80.1% decrease Non-HDL-C: 21.7% decrease ApoB: 0.7% decrease LDL-C: 78.2% increase HDL-C: 67.6% increase RC: 57% decrease
PALISADE Phase 3 (Watts et al., 2025)	2025	Plozasiran	75 patients with TG > 1,000 mg/dL, FCS, low LPL activity or a history of acute pancreatitis	78% decrease with 50 mg at 10 months	APOC-III: 93% decrease Non-HDL-C: decrease from 271.7 to 150.5 mg/dL ApoB: increase from 69.7 to 72.6 mg/dL LDL-C: increase from 25.8 to 51.9 mg/dL HDL-C: increase from 14.6 to 26 mg/dL

Abbreviations: APOC-III, apolipoprotein C-III; TG, triglyceride; LDL-C, low-density lipoprotein cholesterol; non-HDL-C, Non-HDL, cholesterol; ApoB, Apolipoprotein B; HDL-C, high-density lipoprotein cholesterol; FCS, familial chylomicronemia syndrome; MCS, multifactorial chylomicronemia syndrome; ASCVD, atherosclerotic cardiovascular disease; LPL, lipoprotein Lipase.

ASOs are short, single-stranded DNA molecules that enter the cell nucleus and bind to a specific complementary region of the target gene’s mRNA, leading to its degradation and thereby blocking protein translation. Volanesorsen is a second-generation ASO that does not contain a GalNAc (N-acetylgalactosamine) conjugate. Compared to first-generation ASOs, it offers enhanced potency, stability, and binding affinity, but requires weekly or bi-weekly subcutaneous injections. Third-generation ASOs incorporate a GalNAc moiety, which binds with high affinity to the asialoglycoprotein receptor (ASGPR) found on hepatocyte surfaces. This modification improves drug potency, stability, and target specificity, allowing for lower doses and less frequent administration (Chebli et al., 2024; Makhmudova et al., 2024). siRNAs are short double-stranded RNA molecules that, once in the cytoplasm of hepatocytes, associate with the RNA-induced silencing complex (RISC), resulting in the degradation of target mRNA and suppression of hepatic protein production. Plozasiran is a GalNAc-conjugated siRNA administered subcutaneously every 3 months. It is specifically designed to target and degrade ApoC-III mRNA, thereby inhibiting the production of ApoC-III protein in the liver (Chebli et al., 2024; Makhmudova et al., 2024).

#### 7.1.1 Volanesorsen

Volanesorsen, the first drug targeting apoC-III using an ASO method, inhibits the production of apo-CIII by targeting the destruction of apoC-III mRNA (Paragh et al., 2022). In the COMPASS trial, 26 weeks of treatment in patients with familial chylomicronemia syndrome (FCS) led to marked reductions in TG (−71.2%), VLDL-C (−71.5%), apo-B48 (−71.1%), non-HDL-C (−27.3%), and chylomicron TG (−78.1%) after 3 months (Chebli et al., 2024). Injection site reactions occurred in 24% of treated patients, and thrombocytopenia was reported in 9 patients, with one case of platelet count <50,000/μL leading to treatment discontinuation (Gouni-Berthold et al., 2021).

The APPROACH trial showed similar results in severe hypertriglyceridemia, with 3-month reductions of apoC-III and TG by 84% and 77%, respectively; 77% of treated patients achieved TG < 750 mg/dL *versus* 10% in placebo. HDL-C and LDL-C levels increased due to enhanced VLDL and IDL conversion (Prohaska et al., 2023). Long-term follow-up indicated sustained TG reduction, though somewhat attenuated over time, with injection site reactions, thrombocytopenia, and fatigue as the most frequent adverse events (Jones et al., 2023; Witztum et al., 2019).

In a randomized, placebo-controlled trial in patients with familial partial lipodystrophy (FPLD), weekly volanesorsen treatment for 3 months resulted in a significant reduction in TGs (−88% vs. −22% with placebo). After 12 months, patients receiving volanesorsen also showed a greater reduction in hepatic fat fraction (HFF) compared with placebo (53% vs. 8%) (Oral et al., 2022).

#### 7.1.2 Olezarsen

Olezarsen is a new N-acetyl-galactosamine (GalNAc)-conjugated ASO that inhibits apoC-III generation by targeting apoC-III mRNA destruction in the nucleus of hepatocytes (Tokgozoglu et al., 2023). In a phase 1/2a double-blind, placebo-controlled, dose-escalation trial in individuals with elevated TGs, single-dose olezarsen produced dose-dependent TG reductions of up to 77% at 2 weeks, with concurrent decreases in VLDL-C, non-HDL-C, and apoB, and increases in HDL-C. In the multiple-dose cohorts, weekly dosing reduced TG by up to 73%, alongside improvements in other lipid parameters (Alexander et al., 2019). Olezarsen was generally well tolerated, with mostly mild adverse events and no treatment-related serious safety concerns reported.

In a randomized, placebo-controlled trial by Tardif et al. including 114 patients with fasting TG levels of 200–500 mg/dL, olezarsen administered over 6 months produced dose-dependent reductions in TG of up to 60%. Significant improvements were also observed in apoC-III (up to −74%), VLDL-C (up to −58%), non-HDL-C (up to −24%), and apoB (up to −17%) (Tardif et al., 2022). Olezarsen was generally well tolerated, with adverse events similar to placebo; most were mild to moderate, and no severe events were considered treatment-related (Tardif et al., 2022).

In a substudy of the ESSENCE-TIMI 73b trial including 468 participants, most had moderate hypertriglyceridemia and received olezarsen as an add-on to lipid-lowering therapy (Marston et al.). At 6 months, olezarsen reduced TGs by 63.9%, remnant cholesterol by 71.9%, and apolipoprotein B by 16.0% *versus* placebo, with minimal change in LDL-C. However, at 12 months, there were no significant differences between groups in low-attenuation, calcified, or total plaque volumes (Marston et al.). In a second substudy of the ESSENCE-TIMI 73b trial, TG reductions with olezarsen were greater in participants using fibrates at baseline. Among 1,349 patients (309 fibrate users), placebo-adjusted TG reduction at 1 year was 76.6% with fibrates *versus* 49.5% without, with greater reductions also observed in apo C-III, non-HDL-C, and total cholesterol (Wang et al., 2026).

Olezarsen significantly reduces TRL particles, including total (−51%), large (−68%), and medium (−63%) TRLs, while favorably shifting LDL particle size by increasing large LDL and reducing small LDL. It also increases total HDL particles, particularly small HDL (+32%), overall suggesting an improved atherogenic profile (Karwatowska-Prokopczuk et al., 2022). Olezarsen has demonstrated robust efficacy in familial chylomicronemia syndrome and hypertriglyceridemia, with a more favorable safety and tolerability profile compared with volanesorsen. Several ongoing phase 3 trials (e.g., BALANCE, CORE, and ESSENCE programs) are further evaluating its efficacy across severe and moderate hypertriglyceridemia and high cardiovascular risk populations (Banach et al., 2023; Gouni-Berthold et al., 2023). Olezarsen is the only treatment approved by the U.S. FDA for FCS as an adjunct to diet (Syed, 2025).

#### 7.1.3 Plozasiran

Plozasiran (ARO-APOC3) is a GalNAc-conjugated siRNA that inhibits hepatic apoC-III synthesis *via* cytoplasmic mRNA degradation. In a phase 1 study (NCT03783377) including patients with hypertriglyceridemia (TG ≥ 300 mg/dL) and multifactorial chylomicronemia (TG ≥ 880 mg/dL), plozasiran reduced apoC-III levels by 96% at week 4. Correspondingly, TG levels decreased by 78% and 92%, respectively, with several patients achieving TG < 150 mg/dL. Significant reductions in non-HDL-C (−64%) and marked increases in HDL-C (+136%) were also observed in the MCM group. The treatment was well tolerated, with no serious adverse events; only transient ALT elevations were reported in two patients (Clifton et al., 2020).

In a 16-week phase 1 study by Clifton et al., plozasiran markedly reduced apoC-III (−98.2%), TG (−91.3%), and non-HDL-C (−58.3%), while increasing HDL-C (+152.4%) in patients with FCS. In patients with MCM, apoC-III (−96%), TG (−89.8%), and non-HDL-C (−48.6%) were similarly reduced, with HDL-C increased by 110.8%. The treatment was well tolerated, with no significant differences in adverse events across dosing groups (Clifton et al., 2021).

In a phase 2b randomized trial (NCT04998201) in patients with mixed dyslipidemia, subcutaneous plozasiran administered quarterly or semi-annually produced significant reductions in fasting triglycerides of up to 62.4% at 24 weeks and 56.7% at 48 weeks compared with placebo. Modest increases in LDL-C were observed, particularly in those with higher baseline triglycerides, and mild worsening of glycemic control was reported at higher doses (Ballantyne et al., 2024).

The SHASTA-2 phase 2b trial (NCT04720534) evaluated plozasiran at doses of 10 mg, 25 mg, and 50 mg administered subcutaneously on day 1 and week 12 in adults with severe hypertriglyceridemia (fasting triglycerides between 500 and 4,000 mg/dL) (Gaudet et al., 2024). Results showed a dose-dependent reduction in triglyceride levels at week 24, with up to a 57% decrease compared to placebo. Additionally, LDL-C levels increased in a dose-dependent manner, significantly rising by 60% in the highest dose group. However, apoB levels remained stable, and apoB-48 and non-HDL cholesterol levels significantly decreased across all doses (Gaudet et al., 2024).

The PALISADE phase 3, randomized, placebo-controlled trial in patients with FCS showed that plozasiran administered every 3 months reduced median TGe levels by up to 80% and apoC-III by up to 96% at 10 months. Treatment was also associated with an 83% reduction in the risk of acute pancreatitis compared with placebo (Watts et al., 2025).

### 7.2 Targeting ANGPTL3

Angiopoietin-like protein 3 (ANGPTL3) is produced in the liver and plays a crucial role in the availability of LDL, TRLs, and HDL by regulating the activity of LPL and endothelial lipase (EL). A number of studies have shown that ANGPTL3 deficiency is associated with a reduced risk of CVD events. Loss-of-function variants in ANGPTL3 are associated with lower values of TG, LDL-C, and HDL-C (Eguchi et al., 2012). Heterozygous carriers of loss of function variants in ANGPTL3 showed a 17% and 12% reduction in TG and LDL-C values, respectively, and a 34% lower odds of coronary artery disease (Dewey et al., 2017) compared with persons with the wild type form of the gene for ANGPTL3. Table 2 provides summary of clinical trials of drugs targeting ANGPTL3.

**TABLE 2:** Clinical trials of drugs targeting ANGPTL3.

Trial	Year	Drug	Mode of action	Population	Duration of treatment	Maximal TG reduction
NCT01749878, phase 1 (Dewey et al., 2017)	2017	Evinacumab	Monoclonal antibody	83 participants with TG 150–450 mg/dL or LDL-C ≥ 100 mg/dL	21 days	76%
NCT01749878, phase 1 (Ahmad et al., 2019)	2019	Evinacumab	Monoclonal antibody	83 participants with TG 150–450 mg/dL or LDL-C ≥ 100 mg/dL	126 days	88%
NCT03175367, phase 2 (Rosenson et al., 2020)	2020	Evinacumab	Monoclonal antibody	272 patients with refractory hypercholesterolemia	4 months	62%
ELIPSE HoFH, phase 3 ((Raal et al., 2020)	2020	Evinacumab	Monoclonal antibody	65 patients with HoFH	6 months	55%
NCT01749878, phase 1 (Ahmad et al., 2021)	2021	Evinacumab	Monoclonal antibody	Seven patients with TG 450–1,500 mg/dL (cohort B), nine patients with LPL pathway sequence variations and TG > 1,000 mg/dL (Cohort C)	126 days	Cohort B: 81.8% Cohort C: 93.2%
IONIS-ANGPTL3-LRx, phase 1 (Graham et al., 2017)	2017	Vupanorsen	GalNAc conjugated ASO	44 participants with TG ≥ 90 mg/dL and LDL-C ≥ 70 mg/dL	15–43 days	63%
Gaudet et al., phase 2 (Gaudet et al., 2020)	2020	Vupanorsen	GalNAc conjugated ASO	105 patients with TG > 150 mg/dL, Type 2 diabetes, and hepatic steatosis	25–27 weeks	44%
TRANSLATE -TIMI 70, phase 2 (Bergmark et al., 2022)	2022	Vupanorsen	GalNAc conjugated ASO	286 patients with Non–HDL-C ≥ 100 mg/dL and TG 150–500 mg/dL	6 months	59%
NCT03747224, phase 1 (Watts et al., 2023)	2023	Zodasiran	GalNAc conjugated siRNA	52 healthy participants with TG >100 mg/dL and LDL-C >70 mg/dL and nine patients with hepatic steatosis	85 days	54.4%
ARCHES-2, phase 2 (Gaudet et al., 2024)	2024	Zodasiran	GalNAc conjugated siRNA	204 patients with TG 150–500 and either an LDL-C ≥70 mg/dL or a non-HDL-C ≥100 mg/dL	3 months	63.1%
Ray et al., phase 1 (Ray et al., 2024)	2024	Solbinsiran	GalNAc conjugated siRNA	40 healthy participants with TG 150–500 and LDL-C ≥70 mg/dL	169 days	73%
NCT05256654, phase 2 (Ray et al., 2025)	2025	Solbinsiran	GalNAc conjugated siRNA	205 patients with TG 150–500 and either an LDL-C ≥70 mg/dL or a non-HDL-C ≥100 mg/dL	9 months	52.5%

Abbreviations: ANGPTL3, Angiopoietin-like protein 3; TG, triglyceride; LDL-C, low-density lipoprotein cholesterol; non-HDL-C, Non-HDL, cholesterol; HOFH, homozygous familial hypercholesterolemia; LPL, lipoprotein lipase activity; ASO, antisense oligonucleotide; GalNAc, N-acetylgalactosamine; siRNA, small interfering RNA.

#### 7.2.1 Evinacumab

Evinacumab, a monoclonal antibody targeting ANGPTL3, is approved for homozygous familial hypercholesterolemia (HoFH) and is administered intravenously at 15 mg/kg monthly (Sosnowska et al., 2022). In a phase 1 randomized, placebo-controlled trial, evinacumab produced rapid reductions in triglycerides of up to ∼80% within days, along with modest decreases in LDL-C. Similar marked TG and VLDL-C reductions were observed in patients with moderate to severe hypertriglyceridemia, although responses were variable in those with very high TG levels. Notably, increases in LDL-C were observed in some patients, likely reflecting enhanced VLDL-to-LDL conversion (Dewey et al., 2017; Ahmad et al., 2021).

Studies have evaluated evinacumab in patients with HoFH. In a single-group, open-label trial of 9 patients receiving maximally tolerated lipid-lowering therapy, evinacumab (250 mg subcutaneous followed by 15 mg/kg IV at 2 weeks) reduced LDL-C, apoB, non-HDL-C, TG, and HDL-C by 49%, 46%, 49%, 47%, and 36%, respectively, at week 4, with no treatment interruptions due to adverse events (Gaudet et al., 2017). In a phase 3, double-blind, placebo-controlled trial of 65 HoFH patients, monthly intravenous evinacumab (15 mg/kg) lowered total cholesterol, LDL-C, non-HDL-C, TG, HDL-C, apoB, apoC-III, and Lp(a) by 47.4%, 47.1%, 49.7%, 55%, 29.6%, 41.4%, 84.1%, and 5.5%, respectively, at week 24, with 84% of patients achieving ≥30% reduction in LDL-C. Two serious adverse events occurred (urosepsis and suicide attempt), and both patients recovered (Raal et al., 2020).

A phase 2 trial evaluated monthly intravenous evinacumab (15 mg/kg for 3 months) in three patient groups (Bae, 2024): FCS with complete LPL loss-of-function (Rinella et al., 2023), MCM with heterozygous LPL mutations, and (Idilman et al., 2016) MCM without LPL mutations. Evinacumab had minimal effect on TG in group 1, but reduced TG by 64.8% and 81.7% in groups 2 and 3, respectively, suggesting that some LPL activity is required for evinacumab to effectively lower TGs (Rosenson et al., 2023).

#### 7.2.2 Vupanorsen

Vupanorsen, a second-generation GalNAc-conjugated ASO targeting ANGPTL3 mRNA, reduces ANGPTL3 synthesis and improves lipid profiles. In a phase 1, double-blind, placebo-controlled trial in healthy subjects, single-dose vupanorsen (20–80 mg) produced modest reductions in ANGPTL3, TG, VLDL-C, LDL-C, and non-HDL-C, while multiple weekly doses over 6 weeks resulted in greater decreases: ANGPTL3 (−84.5%), TG (−63.1%), VLDL-C (−60%), LDL-C (−32.9%), non-HDL-C (−36.6%), apoB (−25.7%), and apoC-III (−58.8%), with no serious adverse events reported (Graham et al., 2017). In patients with fasting TG > 150 mg/dL, hepatic steatosis, and type 2 diabetes, subcutaneous vupanorsen for 6 months (20–80 mg weekly) reduced TG by 36%–53%, with the 80 mg dose also lowering total cholesterol (−19%), non-HDL-C (−18%), HDL-C (−24%), apoB (−9%), apoC-III (−58%), and remnant cholesterol (−38%), without serious adverse events (Gaudet et al., 2020).

Treatment with vupanorsen was shown to be associated with dose-response reductions in the levels of non-HDL-C and TG; reductions in the levels of LDL-C and apoB were not dose-dependent. It is noteworthy that vupanorsen caused a dose-dependent increase in HFF, with a 21%, 40% and 76% elevation in HFF at the 80 mg, 120 mg, and 160 mg every 2 weeks, respectively, which led to discontinuation of the study (Bergmark et al., 2022). The development of vupanorsen was stopped after evaluations revealed that its overall benefit did not outweigh the potential risks.

#### 7.2.3 Zodasiran

Zodasiran (ARO-ANG3) is a GalNAc-conjugated siRNA targeting ANGPTL3. In a phase 1 trial in healthy participants and individuals with hepatic steatosis, zodasiran was well tolerated and produced reductions in triglycerides (up to 58.6%), LDL-C (up to 24.2%), and non-HDL-C (26.2%) (Watts et al., 2023). In the phase 2 ARCHES-2 trial of 204 patients with mixed hyperlipidemia, subcutaneous zodasiran led to dose-dependent TG reductions at 24 weeks of 51%, 57%, and 63% for 50, 100, and 200 mg, respectively, with 88% of patients at the highest dose achieving TG < 150 mg/dL. These improvements were accompanied by decreases in ANGPTL3, LDL-C, non-HDL-C, HDL-C, remnant cholesterol, lipoprotein(a), and apoB, although LDL-C reductions were less pronounced in patients with very high baseline TGs (Rosenson et al., 2024).

#### 7.2.4 Solbinsiran

Solbinsiran (LY3561774) is a GalNAc-conjugated siRNA targeting ANGPTL3. In a phase 1 trial of 40 participants with elevated TG and LDL-C, solbinsiran produced dose-dependent reductions in ANGPTL3 (up to 86%), TG (up to 73%), non-HDL-C (up to 46%), and apoB (up to 36%), with effects lasting up to 169 days and mostly mild adverse events (Rosenson et al., 2024; Ray et al., 2024). In a 270-day phase 2 trial of 205 adults with mixed dyslipidemia, solbinsiran again reduced ANGPTL3 and TG (up to 52.5%) in a dose-dependent manner, with modest LDL-C reductions and durable effects across doses; HDL-C decreased at higher doses, and responses were variable (Ray et al., 2025).

### 7.3 Targeting apolipoprotein C2

Apolipoprotein C2 (apoC-II), which is primarily found on TRL, is an activator of LPL (Mehta and Shapiro, 2022; Tall et al., 2022). ApoC-II is a rate-limiting factor in LPL-mediated lipolysis, with both low and excessively high plasma levels linked to hypertriglyceridemia (Baass et al., 2020; Ueda et al., 2017; Yamamoto et al., 2014). In a cohort study involving 3,141 participants followed over a decade, Silbernagel et al. reported a potential inverse J-shaped association between apoC- II quintiles and CVD mortality. Complementary *in vitro* experiments revealed that LPL activity increased with the addition of exogenous apoC-II, but declined when apoC-II levels became excessively high, again mirroring an inverted J-shaped pattern (Silbernagel et al., 2023). This non-linear relationship and the narrow therapeutic window complicate the potential of apoC-II as a pharmacologic target. Moreover, genome-wide association and Mendelian randomization studies have not provided strong evidence for a causal role of apoC-II in ASCVD (Teslovich et al., 2010). Efforts to develop apoC-II mimetic therapies have so far not advanced beyond the preclinical stage (Wolska et al., 2021).

### 7.4 Analog of FGF21

FGF21 is an endogenous stress-response hormone that plays a key role in regulating lipid and glucose metabolism as well as energy expenditure. Preclinical studies indicate that in the liver, FGF21 reduces fat accumulation by enhancing adenosine monophosphate-activated protein kinase (AMPK) activity. This leads to increased fatty acid oxidation, decreased DNL, and enhanced secretion of VLDL triglycerides, thereby helping to clear existing hepatic fat. In adipose tissue, FGF21 enhances insulin sensitivity and promotes the turnover of TRLs, such as VLDL, by stimulating brown fat activation and inducing the browning of white adipose tissue through upregulation of uncoupling protein 1 (UCP1) (Kliewer and Mangelsdorf, 2019; Lin et al., 2017; Tillman and Rolph, 2020). Additionally, FGF21 has been shown to upregulate LDLR expression, potentially facilitating the clearance of VLDL remnants *via* the ApoE-LDLR pathway (Liu et al., 2022). The phase I trial of pegozafermin included 58 healthy volunteers. Single doses as high as 78 mg resulted in significant improvements in lipid profiles (Rosenstock et al., 2023). Notably, doses of 9.1 mg or higher produced marked changes in triglycerides, LDL-C, and HDL-C levels at both 8 and 15 days after administration. On average, triglycerides decreased by up to 51% (*versus* only 2% with placebo), LDL-C was reduced by as much as 37%, and HDL-C levels increased by up to 36% (Rosenstock et al., 2023). In a phase 2, double-blind, randomized trial, pegozafermin was evaluated in 67 patients with severe hypertriglyceridemia (TGs ≥500 mg/dL and ≤2,000 mg/dL) across four dosing arms and compared with 18 patients receiving placebo over 8 weeks. The pooled pegozafermin group showed a significant median triglyceride reduction of 57.3% *versus* 11.9% with placebo, resulting in a placebo-adjusted difference of −43.7%. TG reductions ranged from 36.4% to 63.4% across treatment arms and were consistent regardless of concomitant lipid-lowering therapy. Secondary outcomes showed significant reductions in apoB (−10.5% vs 1.1%) and non-HDL-cholesterol (−18.3% vs −0.6%) compared with placebo. No serious adverse events were attributed to the drug (Bhatt et al., 2023). The phase 3 ENTRUST trial (NCT05852431), comparing pegozafermin to placebo in patients with severe hypertriglyceridemia over 26 weeks, is currently enrolling participants (Hartsfield et al., 2024).

### 7.5 Targeting DGAT2

DGAT2 inhibition is considered as a promising therapeutic strategy for metabolic diseases characterized by hepatic lipid accumulation, with secondary benefits on circulating TG levels. Three Phase I studies evaluated the safety and tolerability of the DGAT2 inhibitor ervogastat, including two studies in healthy adult participants and one in adults with MASLD (Amin et al., 2023). In healthy participants, repeated escalating doses of ervogastat resulted in a general reduction in fasting serum TGs compared with placebo (−8.2% to −29.7%), although postprandial triglyceride changes did not show a consistent dose–response relationship. In participants with MASLD, 14 days of ervogastat treatment produced dose-dependent reductions in liver fat (−24.3% and −33.9% for 50 and 300 mg, respectively) and decreased fasting and postprandial TGs *versus* placebo. However, TG reductions were not strictly dose dependent. safety data showed a high frequency of gastrointestinal adverse events, increasing with dose (≥3 mg) and exceeding placebo at all doses. Diarrhea was the most common event (38.5%), followed by nausea, flatulence, vomiting, and abdominal pain (Amin et al., 2023).

## 8 TG-lowering treatments and hepatic steatosis

Several triglyceride-lowering treatments have been investigated for their effects on MASLD (Table 3).

**TABLE 3:** Summary of triglyceride-lowering treatments investigated for their effects on steatosis, steatohepatitis, and liver fibrosis.

Drug/class	Mechanism(s)	Steatosis	Steatohepatitis	Liver fibrosis
Statins	Inhibit HMG-CoA reductase, reducing cholesterol synthesis in the liver	Reduce development of hepatic steatosis	Improves histological features of steatohepatitis	Improvement in hepatic fibrosis
Fibrates (Fenofibrate)	Stimulate PPARα	No significant reduction	No effect	No effect
Fibrates (Pemafibrate)	Stimulate PPARα	Reduces liver fat accumulation	Reduces inflammation	Improvement in hepatic fibrosis
Omega-3 fatty acids (EPA-EE)	Mechanism uncertain	No effect	No effect	No effect
PCSK9 inhibitors	Enhance LDL receptor activity and modulate remnant lipoproteins	Reduce or completely resolve hepatic steatosis	May improve inflammation and liver injury markers (preclinical and some clinical evidence)	May improve fibrosis (preclinical evidence; clinical effect not fully defined)
Volanesorsen	Inhibits ApoC-III, enhancing LPL activity and redirecting FFAs from liver to adipose tissue	Reduces HFF in FPLD, FCS, and severe hypertriglyceridemia	Not evaluated	Not evaluated
Vupanorsen	Inhibits ANGPTL3	No reduction; may increase HFF	Not evaluated	Not evaluated
DGA2 inhibitors	Inhibits DGAT2, blocking the final step of triglyceride synthesis and reducing hepatic triglyceride formation and lipid droplet accumulation	Reduces hepatic steatosis	Improves histological features of steatohepatitis; combination therapy (ervogastat + clesacostat) shows higher rates of Steatohepatitis resolution compared with monotherapy	Improvement in hepatic fibrosis, particularly with combination therapy (ervogastat + clesacostat).
Solbinsiran	Mechanism uncertain	Reduces HFF in adults with mixed dyslipidemia	Not evaluated	Not evaluated

Abbreviations: HMG-CoA, 3-hydroxy-3-methylglutaryl-coenzyme A; PPARα, peroxisomal proliferator-activated receptor alpha; EPA-EE, EPA, ethyl ester; PCSK9, proprotein convertase subtilisin/kexin type 9; LDL, low-density lipoprotein; APOC-III, apolipoprotein C-III; LPL, lipoprotein lipase; HFF, hepatic fat fraction; FPLD, familial partial lipodystrophy; FCS, familial hyperchylomicronaemia syndrome; ANGPTL3, angiopoietin-like 3.

### 8.1 Statins

Statins may have the potential to impact MASLD/MASH since they have pleiotropic effects apart from their lipid-lowering. Statins have the capacity to mitigate hepatic lipotoxicity, inflammation, oxidative stress, and fibrosis arising from MASH *via* different mechanisms (Dehnavi et al., 2021; Tzanaki et al., 2022). Statin treatment is associated with increases in the levels of paraoxonase 1 (PON1), an antiatherogenic and antioxidant enzyme that is reduced in those with MASLD. In MASLD, the levels of PPARα is reduced, which can be enhanced following statin treatment, restoring mitochondrial fatty acid oxidation (FAO) (Ahsan et al., 2020; Gluba et al., 2010). Simvastatin may help inhibit fibrosis progression in MASH patients by downregulating the expression of α-smooth muscle actin (α-SMA) (0.79- and 0.86-fold), Collagen I (0.79- and 0.82-fold), and inducible nitric oxide synthase (iNOS) (0.30- and 0.45-fold), while upregulating endothelial nitric oxide synthase (eNOS) expression (2.18- and 1.73-fold), at both mRNA and protein levels, as measured by real-time reverse transcriptase-polymerase chain reaction and Western blot analysis (Wang et al., 2013). These pleiotropic effects translate into clinically relevant benefits across key histological features of MASLD.

Several systematic reviews, meta-analyses, and large population-based studies consistently indicate that statin use is associated with a favorable MASLD prognosis (Fatima et al., 2022; Pastori et al., 2022; Rattanachaisit et al., 2018). Statin therapy has been linked to a reduced risk of developing MASLD, as well as a lower likelihood of disease progression to advanced fibrosis and cirrhosis. In addition, statin use has been associated with decreased liver-related complications and overall mortality, suggesting a broader beneficial effect on long-term clinical outcomes in patients with MASLD (Fatima et al., 2022; Pastori et al., 2022; Rattanachaisit et al., 2018).

Regarding hepatic steatosis, a Cochrane meta-analysis reported improvements in sonographic steatosis, although the included trials were small and at high risk of bias (Eslami et al., 2013). One randomized trial demonstrated a 71% reduction in the odds of hepatic steatosis with a combination of atorvastatin and vitamins C and E over 4 years, although the independent contribution of the statin cannot be determined (Foster et al., 2011). Despite the majority of evidence suggesting a beneficial effect of statins on hepatic steatosis, two randomized controlled trials did not demonstrate improvement, including one small, high-risk-of-bias study (Nelson et al., 2009) and another that used a lower-potency statin, pitavastatin (Braun et al., 2018).

Beyond steatosis, statins have also been associated with improvements in steatohepatitis and fibrosis. A large nationwide cohort from Korea, including over 11 million individuals, showed that statin use was associated with a lower risk of developing MASLD and, among those with established disease, a reduced risk of significant liver fibrosis (OR: 0.43) (Lee et al., 2021). Similarly, a multicenter European cohort of 1,201 individuals demonstrated lower odds of MASH and reduced fibrosis stages in statin users compared with non-users (Eguchi et al., 2012). In a clinical trial by Sfikas et al. involving 604 participants with MASLD/MASH, treatment with atorvastatin, rosuvastatin, or pitavastatin for 12 months, alongside diet and exercise, resulted in significant improvements in both MASLD activity and liver fibrosis compared with lifestyle intervention alone (Sfikas et al., 2021).

Overall, these findings suggest that statins may confer benefits across the spectrum of MASLD, although further studies are needed to clarify their long-term impact.

### 8.2 Fibrates

#### 8.2.1 Fenofibrate

The inhibitory effects of fibrates on inflammatory cytokines may provide a mechanism for impacting MASLD/MASH (Kostapanos et al., 2013; Tanaka et al., 2017). Although fibrates were expected to mitigate MASLD/MASH, clinical studies have yet to demonstrate this. A randomized, controlled trial evaluated the effects of fenofibrate (200 mg/d for 2 months) and niacin (2000 mg/dL for 4 months) on intrahepatic triglyceride content. While both treatments reduced plasma levels of VLDL-TG, neither reduced intrahepatic fat content (Fabbrini et al., 2010b). In another trial of 78 obese patients with MASLD, 3 months of treatment with fenofibrate and omega-3 carboxylic acid (OM-3CA) reduced serum TG levels significantly; however, neither treatment reduced hepatic fat significantly (Oscarsson et al., 2018). Fenofibrate also failed to show protective effects on liver pathology (lobular inflammation, grade of steatosis, and liver fibrosis) of patients with MASLD (Fernández-Miranda et al., 2008).

#### 8.2.2 Pemafibrate

In mouse models of MASLD/MASH, pemafibrate was shown to lower liver enzyme levels and improve key pathological features such as hepatic steatosis, ballooning, inflammation, and fibrosis (Honda et al., 2017; Kanno et al., 2022; Sasaki et al., 2020; Takei et al., 2017). Mechanistically, pemafibrate appears to impact MASLD by upregulating genes involved in hepatic β-oxidation and lipid export, as well as enhancing overall energy metabolism through induction of the uncoupler protein 3 (UCP3) gene. Kanno et al. investigated effects of pemafibrate in a novel mouse model of diet-induced steatohepatitis-related cardiomyopathy using a high-fat, high-cholesterol, high-sucrose, and bile acid-rich diet (MASH diet) (Kanno et al., 2022). Mice were fed this diet for 8 weeks, with or without pemafibrate (0.1 mg/kg). Compared with the control diet group, mice on the MASH diet exhibited increased hepatic macrophage infiltration, fibrosis, and steatohepatitis characterized by elevated free cholesterol and cholesterol crystal accumulation. Cardiac involvement included free cholesterol deposition, concentric hypertrophy, and reduced left ventricular ejection fraction. Additionally, activation of the nucleotide-binding oligomerization domain (NOD)-like receptor and PI3K-Akt pathways was observed, along with increased expression of inflammasome-related genes such as caspase-1, nucleotide-binding oligomerization domain leucine rich repeat and pyrin domain containing 3 (NLRP3), and IL-1β in both liver and heart tissue. These findings indicated that pemafibrate may mitigate liver fat accumulation, inflammation, fibrosis, and cardiac dysfunction, even after the onset of steatohepatitis-related heart disease (Kanno et al., 2022).

To explore the clinical relevance of these findings, several retrospective single-arm studies in Japan assessed the effectiveness of pemafibrate in small cohorts of MASLD patients (Hatanaka et al., 2021a; Hatanaka et al., 2021b; Ikeda et al., 2020; Ikeda et al., 2021; Shinozaki et al., 2020). Despite some variability, these studies consistently reported improvements in liver enzymes (ALT, alkaline phosphatase (ALP), gamma-glutamyl transferase (GGT)) and fibrosis indicators such as the AST-to-platelet ratio index and the FIB-4 index. A prospective single-arm study involving 20 MASLD patients with dyslipidemia administered pemafibrate (0.1 mg twice daily) for 12 weeks was performed (Seko et al., 2020). The primary outcome was the change in serum ALT from baseline to week 12, which showed a significant reduction. Additionally, improvements were observed in serum levels of triglycerides, HDL-cholesterol, total fatty acids, saturated fatty acids, and unsaturated fatty acids (Seko et al., 2020).

A phase 2 randomized trial in Japan tested pemafibrate (0.2 mg twice daily for 72 weeks) in patients with MASLD/MASH (Nakajima et al., 2021). While it did not significantly reduce liver fat content, it significantly improved liver stiffness, ALT, and LDL-C levels (Nakajima et al., 2021). These results suggest pemafibrate may help reduce liver fibrosis and could be effective in combination with agents that target liver fat. In rodent models, the combination of pemafibrate with tofogliflozin, a highly selective sodium-glucose cotransporter-2 (SGLT2) inhibitor, significantly prevented the development of MASH (Murakami et al., 2022). A related clinical trial (NCT05327127) is currently underway to evaluate the efficacy of extended release pemafibrate combined with tofogliflozin in patients with MASH, with results expected in late 2026.

### 8.3 Omega-3 fatty acids

It was hypothesized that treattment with ω-3 Polyunsaturated Fatty Acids (PUFAs) may ameliorate MASLD since reduced levels of PUFAs were detected in MASLD patients and, moreover, PUFAs can decrease liver inflammation and lipogenesis (Spadaro et al., 2008). A clinical trial assessed the efficacy of 2 months treatment with the combination of PUFAs and probiotics among 48 MASLD patients with diabetes. The findings showed reductions in systemic inflammation, TG (by 0.75 ± 0.79 mmol/L), total cholesterol (by 0.41 ± 0.54 mmol/L), and fatty liver index (FLI), which decreased from 83.53 ± 2.60 to 76.26 ± 2.96. Additionally, HDL-C levels increased by 0.14 ± 0.32 mmol/L (Kobyliak et al., 2018). Another study reported improvement in lipid profile and carotid intima-media thickness, and attenuation of hepatic steatosis of 20 children with severe MASLD treated with n-3 PUFA for 6 months (Spahis et al., 2018). The 12 months treatment with PUFAs also promoted reductions in AST (by 2 ± 4 IU/L), ALT (by 4 ± 8 IU/L), GGT (by 4 ± 13 IU/L), TG (by 46 ± 88 mg/dL), and fasting glucose (by 6 ± 14 mg/dL) (Capanni et al., 2006). A recent *in vivo* study demonstrated that both ethyl-eicosapentaenoic acid triacylglycerides (EPA-TAG) and EPA ethyl ester (EPA-EE) helped reduce hepatic steatosis by decreasing food intake and lipogenesis, enhancing lipid and phospholipid polyunsaturated fatty acid remodeling, stimulating ketogenesis, and promoting fatty acid oxidation. These effects are likely mediated through activation of the AMPK/adipocytokine signaling pathway. EPA’s beneficial impact was also linked to modulation of gut microbiota composition (Fu et al., 2021). Notably, EPA-TAG showed greater efficacy than EPA-EE in improving high-fat diet-induced MASLD symptoms (Feng et al., 2024). However, a phase 2 trial demonstrated that EPA-EE had no significant effect on the histologic features of MASH, associated markers of IR, inflammation, or hepatic injury and fibrosis (Sanyal et al., 2014).

### 8.4 PCSK9 inhibitors

PCSK9 may play important roles in MASLD pathogenesis. Increased plasma and hepatic levels of PCSK9 were shown to be associated with increased liver TG content and hepatic fatty acid (Theocharidou et al., 2018).

There is an ongoing debate regarding the effects of PCSK9 inhibitors on MASLD (Momtazi-Borojeni et al., 2022). Experimental studies found opposite findings regarding the role of PCSK9 in MASLD and MASH progression. PCSK9 deficit mice showed amelioration (Lebeau et al., 2019a; Zaid et al., 2008) and exacerbation (Kucsera et al., 2021; Lebeau et al., 2019b) of hepatic steatosis under MASLD- and MASH-inducing challenges, respectively. The progression of MASLD is slowed by the delivery of neutrophil-derived microRNA-223 (miR-223) into liver cells through extracellular vesicles that rely on LDLR and ApoE for uptake. Alirocumab increases LDLR protein levels in liver cells, which boosts the transfer of miR-223 and enhances its protective effect (He et al., 2021). Similarly, alirocumab treatment significantly increased LDLR levels and miR-223 expression in the livers of both methionine/choline deficiency-fed and control diet mice, compared with their respective untreated group (He et al., 2021). Furthermore, alirocumab treatment significantly reduced key MASH features caused by the methionine/choline deficiency diet, including steatosis, fibrosis, liver neutrophil and macrophage infiltration, serum ALT level, and MASLD activity score. In addition, it increased expression of a VLDL-related gene, suggesting that alirocumab-induced LDLR upregulation may help protect the liver by promoting VLDL production (He et al., 2021).

In contrast, clinical studies demonstrated that treatment with PCKS9 inhibitors is associated with lower risk of MASLD (Momtazi-Borojeni et al., 2022). A retrospective cohort study of 29 patients with MASLD who were treated with PCKS9 inhibitors, found complete resolution of MASLD radiological characteristics in 8 of 11 participants with hepatic steatosis. Furthermore, both ALT and AST reduced significantly following the treatment (Shafiq et al., 2020). Another observational research among 26 patients with FH, MASLD, and high levels of LDL-C despite receiving maximally tolerated statin and ezetimibe, PCSK9 inhibitors was shown to be efficient in terms of reducing hepatic steatosis biomarkers, in particular in those with low TG/HDL (Scicali et al., 2021). A year-long study on high-risk FH patients found that PCSK9 inhibitor therapy not only lowered LDL-C and cardiovascular risk but also significantly improved liver health, normalizing liver enzymes and resolving signs of MASLD/MASH (Dimakopoulou et al., 2018). In support of this, a 6-month study in FH patients with MASLD showed that adding PCSK9 inhibitors improved liver fat and inflammation markers, especially in those with low TG/HDL ratios. While LDL-C levels remained similar, TRLs and TG/HDL ratios dropped significantly, suggesting PCSK9 inhibitors may help reduce liver fat in certain patients (Scicali et al., 2021).

While current clinical studies suggest that circulating PCSK9 inhibitors may help improve MASLD, more research is needed to assess the impact of intracellular PCSK9 inhibitors like inclisiran, a liver-targeted siRNA that suppresses PCSK9 expression (Macchi et al., 2019). Given its liver-specific action and long duration, monitoring liver safety is essential. Encouragingly, an open-label study in patients with mild to moderate liver impairment found that inclisiran’s safety profile, including liver enzyme and bilirubin levels, was similar to that of individuals with normal liver function (Kallend et al., 2022).

### 8.5 Volanesorsen

Prohaska et al. designed a study to assess the impact of volanesorsen on HFF, which was measured using MRI, in three randomized trials with volanesorsen in patients with FCS (APPROACH), FPLD (BROADEN), and severe hypertriglyceridemia (TG > 500 mg/dL) (COMPASS). In the trial of patients with severe hypertriglyceridemia, after 6 months of treatment, the absolute HFF was reduced by −3.02% (95% CI, [−5.60, −0.60]) relative to the placebo group. After 12 months of treatment, FCS patients show a lower absolute HFF (1.0% [−2.9, 0.0]) compared with the placebo group; however, the difference was not statistically significant. The decrease for patients with FPLD was −8.34% (−13.01, −3.67) after 12 months of treatment. Placebo-adjusted % change from baseline for patients of COMPASS, APPROACH, and BROADEN were −24.2%, −37.1%, and 52.7%, respectively. Moreover, significant correlations between HFF at baseline and absolute change in HFF were detected in all three groups received volanesorsen; however, the correlation between baseline HFF and HFF change was not significant in either of the study groups (Prohaska et al., 2023).

Lightbourne et al. enrolled five subjects with partial lipodystrophy in a 16-week placebo-controlled, randomized, double blind study of 300 mg volanesorsen. After 4 months of treatment, liver TG content decreased in all patients; only one patient showed abnormal levels of liver TG in 16 weeks. Moreover, reductions in beta-hydroxybutyrate, a surrogate of liver FFA oxidation, were observed in all patients (Lightbourne et al., 2022).

Volanesorsen may improve liver fibrosis by suppressing apoC-III, which enhances LPL activity and redirects free fatty acids from the liver to adipose tissue. This likely reduces liver fat accumulation, as supported by decreased HFF, and is not explained by increased fat oxidation (Lightbourne et al., 2022).

### 8.6 Vupanorsen

A double-blind, placebo-controlled trial was conducted to evaluate the efficacy of vupanorsen in patients with fasting TG > 150 mg/dL, type 2 diabetes, and hepatic steatosis. Patients received vupanorsen for 6 consecutive months at dosage of 40 or 80 mg monthly or 20 mg weekly. HFF was reduced by −0.71 (−3.21, 1.78), 2.39 (−0.16, 4.94), −0.12 (−2.87, 2.64), and −1.69 (−4.09, 0.71) in the patients received 40 mg, 80 mg, 20 mg, and placebo, respectively. The corresponding values for least squared mean (LSM) change of HFF was 0.98 (−2.48, 4.44), 4.09 (0.58, 7.59), and 1.57 (−2.10, 5.25), respectively, compared with the control group. Fatty liver index (FLI) was reduced by −6.08 (−11.08, −1.09), −9.21 (−13.99, −4.44), −8.07 (−13.06, −3.07), and −3.50 (−8.21, 1.22) in the patients received 40 mg, 80 mg, 20 mg, and placebo, respectively. The corresponding values for LSM change of FLI was −2.59 (−9.49, 4.31), −5.71 (−12.43, 1.00), and −4.57 (−11.45, 2.32), respectively. Significant elevations in the level of AST and ALT were observed in patients received 20 and 80 mg. Collectively, all treatments caused increase in HFF and neither was able to reduce FLI significantly (Gaudet et al., 2020).

A phase 2b, multicenter, randomized, double-blind, placebo-controlled, dose-ranging, 8-arm parallel-group study of vupanorsen on 286 subjects with TG higher than 150 mg/dL and non-HDL-C≥100 mg/dL reported dose dependent enhance in HFF. HFF relative changes following every 4 weeks vupanorsen at the dosage of 80, 120, and 160 mg were 1.13 (0.95–1.34), 1.24 (1.02–1.51), and 1.24 (1.09–1.41). The corresponding values for every 2 weeks vupanorsen were 1.21 (1.06–1.38), 1.40 (1.23–1.59), and 1.76 (1.51–2.05), respectively. The median HFF at baseline was higher in those with diabetes and those with BMI or baseline TG equal or higher than median. Treatment enhanced AST and ALT levels in a dose-dependent manner. Elevations in AST and ALT more than three times the upper limit of normal were more prevalent in those received the treatment compared with the placebo group. 44.4% of those received 160 mg vupanorsen every 2 weeks experienced elevated liver functions tests and tests remained elevated in 38.9% for a week. Importantly, a moderate positive correlation between AST and ALT and absolute change in HFF were detected; however, the sensitivity of liver functions tests for detecting changes in HFF was low, indicating that they cannot be used as valuable indicators to detect change in HFF (Bergmark et al., 2022; Zimerman et al., 2023). These side effects prompted discontinuation of the vupanorsen program (Kosmas et al., 2022).

In contrast to clinical studies, experimental studies found promising findings about the effects of ANGPTL3 inhibition on hepatic steatosis. An *in vivo* study showed that treatment with ANGPTL3 ASO resulted in an 81% significant reduction in TG content of the liver compared with the control group (8.7 ± 2.6 and 45.5 ± 14.6 mg per gram in the treatment and control groups, respectively) (Graham et al., 2017). Similarly, a vaccine targeting ANGPTL3 was shown to reduce liver TG content significantly and after 6 weeks of treatment, histological examination demonstrated that the treatment mitigated liver steatosis, lobular inflammation, and hepatocyte ballooning. Additionally, treatment contributed to reductions in the levels of FASN and ACC1, which are responsible for FAA synthesis in the liver (Fukami et al., 2021). Verve Therapeutics reported preclinical findings on VERVE-201, a lipid nanoparticle (LNP)-delivered adenine base editor designed to disrupt the *ANGPTL3* gene by introducing a premature stop codon. In studies involving both wild-type nonhuman primates (NHPs) and an NHP model of HoFH, administration of the NHP-specific version (VERVE-201cyno) led to an approximately 88% reduction in circulating ANGPTL3 levels 90 days post-treatment (Khera et al., 2022). A recent study using a mouse model (LDLR knockout) treated with the murine version (VERVE-201mu) demonstrated an average 47% reduction in LDL-C, along with a decrease in blood triglyceride levels (72%). In NHPs, liver editing of *ANGPTL3* reached up to 63%, and ANGPTL3 protein levels dropped by 95% within 7 days following a 3.0 mg/kg dose. Notably, animals showed no signs of liver toxicity following treatment with the LNP-based therapy (Lee et al., 2023).

Determining the effect of vupanorsen on hepatic steatosis is of great importance since a number of novel treatments targeting ANGPTL3 are in development. It is not completely clear why the treatment was not able to affect hepatic steatosis in a positive manner in opposite to experimental studies, but the difference can be attributed to marked baseline hepatic steatosis of patients (17.6%) or lower decrease in ANGPTL3 levels in the clinical studies compared with experimental studies. So far, it is not fully clear that this effect is an off-target effect of the drug due to intracellular ANGPTL3 inhibition or the metabolic effects of the drug; however, due to existing experimental findings, probably this effect is not because of intracellular ANGPTL3 inhibition.

### 8.7 Pegozafermin

A previous genome-wide meta-analysis involving 33,000 participants identified the rs838133 variant in the FGF21 gene as being linked to reduced protein intake and increased carbohydrate consumption, which may influence dietary macronutrient preferences and contribute to metabolic dysfunction-associated steatotic liver disease development (Romeo et al., 2008). Further analysis in a separate cohort showed that this variant was associated with greater consumption of sweet foods like candy and elevated plasma FGF21 levels following oral sucrose intake, indicating that the liver might release hormones that affect eating behavior (Søberg et al., 2017). Additionally, the A-allele of rs838133 has been strongly correlated with higher blood pressure and waist-to-hip ratio, despite being linked to a lower overall body fat percentage (Anstee et al., 2020), and is also associated with higher salt intake (Saber-Ayad et al., 2020). More recently, the minor A-allele has been connected to increased liver inflammation and significant and advanced fibrosis in metabolic dysfunction-associated steatotic liver disease patients (Bayoumi et al., 2021; Gallego‐Durán et al., 2024).

Several studies have also linked SNPs in the FGF21 gene region to behaviors such as smoking, dietary habits, and alcohol intake (Barros and Hegele, 2025), suggesting an influence on lifestyle choices that may indirectly contribute to the risk of dyslipidemia and metabolic dysfunction-associated steatotic liver disease (Jensen et al., 2018; Okamoto et al., 2018; Yuan et al., 2022). These associations are thought to arise from changes in FGF21 activity within the central nervous system, as FGF21 is capable of crossing the blood-brain barrier and exerting central effects. Animal studies further support this by showing that FGF21 can reduce alcohol and sugar consumption through signaling in the hypothalamus and hindbrain (Jensen-Cody et al., 2020; Schumann et al., 2016; von Holstein-Rathlou et al., 2016). Therefore, even subtle genetic variations in FGF21 may influence key physiological and behavioral pathways involved in lipid metabolism, potentially promoting or protecting against metabolic disorders.

Pegozafermin was designed as a therapeutic agent for managing MASH and severe hypertriglyceridemia (Bhatt et al., 2023; Loomba et al., 2022; Loomba et al., 2023a; Loomba et al., 2019; Loomba et al., 2023b; Rosenstock et al., 2019). A phase 1b/2a randomized, double-blind, placebo-controlled trial evaluated multiple ascending doses of pegozafermin in adults with biopsy-confirmed MASH. By week 13, pegozafermin significantly reduced HFF across all doses and improved liver enzymes and lipid parameters (triglycerides, LDL-C, HDL-C, non-HDL-C) at select doses. Additional benefits included increased adiponectin, reduced PRO-C3, and modest weight loss. No significant changes were observed in insulin resistance or HbA1c. The most common adverse event was mild increased appetite, not associated with weight gain. Two participants discontinued due to adverse events, and no treatment-related serious adverse events or deaths occurred (Loomba et al., 2023a). In another phase 2 randomized trial of pegozafermin for MASH, the percentage of patients achieving MASH resolution was significantly higher than placebo, reaching up to 37% in the 15 mg group, 23% in the 30 mg group, and 26% in the 44 mg group, compared with just 2% with placebo (Loomba et al., 2023b). Fibrosis improvement was also more common with pegozafermin treatment. The most frequent adverse events were nausea and diarrhea.

### 8.8 DGAT2 inhibitors

Ervogastat (PF-06865571) is a first-in-class, orally administered DGAT2 inhibitor that has demonstrated dose-dependent reductions in liver fat, and reductions in serum TGs, following 2-week dosing (Amin et al., 2023). In two parallel phase 2a randomized, placebo-controlled trials in adults with MASLD, ergovastat monotherapy reduced hepatic steatosis by −35.4% at week 6, while co-administration with clesacostat (ACC inhibitor) achieved a greater reduction of −44.6%; adverse events occurred in 36% of patients, with no treatment-related discontinuations (Calle et al., 2021).

In the MIRNA phase 2 randomized controlled trial, ergovastat monotherapy did not achieve the composite primary endpoint (MASH resolution without fibrosis worsening, at least one stage fibrosis improvement without MASH worsening, or both), with response rates of 39% (150 mg) and 45% (300 mg). In contrast, both combination therapy arms with clesacostat met the endpoint, with higher response rates of 66% (150 mg + 5 mg) and 63% (300 mg + 10 mg) (Wong et al., 2025). Across all ergovastat monotherapy doses, there were no clear differences from placebo in fasting serum TGs or other lipid parameters, indicating a neutral effect on circulating lipids despite reductions in hepatic steatosis.

Another DGAT2-targeting therapy, ION224, is a liver-directed antisense oligonucleotide evaluated in MASLD. In a phase 2 randomized trial, the primary endpoint, defined as a ≥2-point reduction in NAFLD Activity Score, with ≥1-point improvement in hepatocellular ballooning or lobular inflammation and no worsening of fibrosis, was achieved in 46% (90 mg) and 59% (120 mg) of patients *versus* 19% with placebo. Only the 120-mg group demonstrated statistically significant improvement in fibrosis improvement (32·4%) compared to placebo (12·5%). There were no deaths and no treatment-related serious adverse events (Loomba et al., 2025).

### 8.9 Solbinsiran

In a 270-day, randomized, double-blind Phase 2 trial of 205 adults with mixed dyslipidemia, solbinsiran administered subcutaneously at 100 mg, 400 mg, or 800 mg (on days 0 and 90) achieved significant reductions in HFF at day 180 (Ray et al., 2025). Compared with placebo, all dosing regimens showed greater declines: −26.8% (100 mg), −42.4% (400 mg), and −35.9% (800 mg). Moreover, HFF reduction with the 400 mg and 800 mg doses was independent of weight loss. Although the reduction in HFF is encouraging, its validation in prospective efficacy trials is essential, especially given the significant overlap in metabolic dysfunction pathways that drive both metabolic dysfunction–associated steatotic liver disease and mixed dyslipidaemia.

## 9 Conclusion

Hepatic steatosis, particularly in the context of MASLD, is increasingly recognized as a key metabolic disorder closely linked to dyslipidemia, insulin resistance, and cardiovascular risk. Triglyceride-lowering therapies have emerged as a promising strategy to mitigate liver fat accumulation and improve metabolic outcomes. Evidence from both clinical and preclinical studies suggests that agents such as fibrates, omega-3 fatty acids, and emerging therapies like PCSK9 inhibitors, pegozafermin, volanesorsen, solbinsiran, and DGAT2 inhibitors, can reduce hepatic triglyceride content and improve liver function markers. However, heterogeneity in patient populations, outcome definitions, and treatment duration limits definitive conclusions regarding long-term benefits, particularly in advanced liver disease. Future trials should focus on robust endpoints, including histological resolution of steatosis and fibrosis progression, and consider patient stratification based on metabolic profiles. Overall, targeting hypertriglyceridemia represents a rational and potentially effective component of the therapeutic approach to hepatic steatosis.

## Glossary

ABCA1: ATP-binding cassette transporter A1
ABCG1: ATP-binding cassette transporter G1
ACC: Acetyl-CoA carboxylase
ACC/AHA: The American College of Cardiology/American Heart Association
ACYL: ATP-citrate lyase
ALA: alpha-linolenic acid
α-SMA: alpha-smooth muscle actin
ALP: alkaline phosphatase
ANGPTL3: angiopoietin-like protein 3
ApoA1: apolipoprotein A-I
ApoC-II: Apolipoprotein CII
ApoC-III: Apolipoprotein C-III
ASCVD: atherosclerotic cardiovascular disease
ASGPR: asialoglycoprotein receptor
ASO: antisense oligonucleotide
CETP: cholesteryl ester transfer protein
ChREBP: carbohydrate response element binding protein
CVD: cardiovascular disease
DAG: diacylglycerol
DGAT2: diacylglycerol acyltransferase 2
DHA: docosahexaenoic acid
DNL: *de novo* lipogenesis
EL: endothelial lipase
eNOS: endothelial nitric oxide synthase
EPA: eicosapentaenoic acid
EPA-EE: EPA ethyl ester
EPA-TAG: ethyl-eicosapentaenoic acid triglycerides
ER: endoplasmic reticulum
ESC/EAS: the European society of Cardiology/the European Atherosclerosis Society
FABP: fatty acid binding protein
FAS: Fatty acid synthase
FATP: fatty acid transporter protein
FCS: familial hyperchylomicronaemia syndrome
FFA: free fatty acid
FGF21: fibroblast growth factor 21
FPLD: familial partial lipodystrophy
GGT: gamma-glutamyl transferase
GISSI-P: Gruppo Italiano per lo Studio della Sopravvivenza nell'Infarto Miocardio – Prevenzione
HFF: hepatic fat fraction
HSL: hormone sensitive lipase
IDL: intermediate density lipoprotein
iNOS: inducible nitric oxide synthase
IR: insulin resistance
JELIS: Japan EPA Lipid Intervention Study
LDL-C: low-density lipoprotein cholesterol
Apob: Apolipoprotein B (apoB)
HDL-C: high-density lipoprotein cholesterol
LPL: lipoprotein lipase
LSM: least squared mean
MASH: metabolic dysfunction-associated steatohepatitis
MASLD: metabolic dysfunction-associated steatotic liver disease
MCM: multifactorial chylomicronemia
NAFLD: non-alcoholic fatty liver disease
NHPs: nonhuman primates
NLRP3: NLR family pyrin domain containing 3
OM-3CA: omega-3 carboxylic acid
PCSK9: proprotein convertase subtilisin/kexin type 9
PPARα: peroxisomal proliferator-activated receptor alpha
PUFAs: Polyunsaturated Fatty Acids
sdLDL: small dense LDL
SFA: saturated fatty acids
SGLT2: sodium-glucose cotransporter-2
siRNA: small interfering RNA
SR-B1: scavenger receptor BI
SREBP-1c: sterol regulatory element binding protein 1c
TG: triglyceride
TM6SF2: transmembrane 6 superfamily member 2
TRLs: Triglyceride-rich lipoproteins
UCP: uncoupling protein 1
UCP3: uncoupler protein 3
UFAs: unsaturated fatty acids
VLDL: very-low-density lipoprotein
