# Correction: Triglyceride-lowering therapies for hepatic steatosis: mechanisms, efficacy, and clinical perspectives

**DOI:** 10.3389/fphar.2026.1943610

**Published:** 2026-07-28

**Authors:** Seyed Saeed Tamehri Zadeh, Peter P. Toth, Maciej Banach

**Affiliations:** 1 International Lipid Expert Panel (ILEP), Lodz, Poland; 2 Preventive Cardiology, CGH Medical Center, Rock Falls, IL, United States; 3 Ciccarone Center for the Prevention of Cardiovascular Disease, Johns Hopkins University School of Medicine, Baltimore, MD, United States; 4 Faculty of Medicine, John Paul II Catholic University of Lublin, Lublin, Poland

**Keywords:** ANGPTL3, ApoC-III, hepatic steatosis, metabolic dysfunction-associated steatotic liver disease, omega-3 fatty acids, pcsk9, statins, triglyceride

Affiliation International Lipid Expert Panel (ILEP), Lodz, Poland was omitted from the paper. This affiliation has now been added for author Seyed Saeed Tamehri Zadeh.

Affiliation Medical School, University of Western Australia, Perth, WA, Australia was erroneously included in the paper. This affiliation has now been removed for author Seyed Saeed Tamehri Zadeh.

The original version of this article has been updated.

